# Supplementary material for: Adaptive resistance of melanoma cells to RAF inhibition via reversible induction of a slowly dividing de‐differentiated state
Source: Mol Syst Biol. 2017 Jan 9;13(1):905. doi: 10.15252/msb.20166796 (PMC5248573; doi:10.15252/msb.20166796)
Supplement: Supplementary file 1 — Appendix [file MSB-13-905-s001.pdf]

Appendix

Adaptive resistance of melanoma cells to RAF inhibition via reversible induction of a slowly-dividing de-differentiated state

Mohammad Fallahi-Sichani[1], Verena Becker[1], Benjamin Izar[2,3], Gregory J. Baker[1], Jia-Ren Lin[4], Sarah A. Boswell[1], Parin Shah[2], Asaf Rotem[2], Levi A. Garraway[2,3,5], and Peter K. Sorger[1,4,5]

[1]Program in Therapeutic Sciences, Department of Systems Biology, Harvard Medical School, Boston, MA
[2]Department of Medical Oncology, Dana–Farber Cancer Institute, Boston, MA
[3]Broad Institute of Harvard and MIT, Cambridge, MA
[4]HMS LINCS Center and Laboratory of Systems Pharmacology, Harvard Medical School, Boston, MA
[5]Ludwig Center at Harvard, Harvard Medical School, Boston, MA

Address correspondence to:
Peter K. Sorger
Tel: +1 617 432 6901; Email: peter_sorger@hms.harvard.edu
Mohammad Fallahi-Sichani
Tel: +1 617 432 6907; Email: mohammad_fallahisichani@hms.harvard.edu

Appendix Figures

Appendix Figure S1	page 2
Appendix Figure S2	page 3
Appendix Figure S3	page 4
Appendix Figure S4	page 4
Appendix Figure S5	page 5
Appendix Figure S6	page 6
Appendix Figure S7	page 7
Appendix Figure S8	page 8

Appendix Figure S1

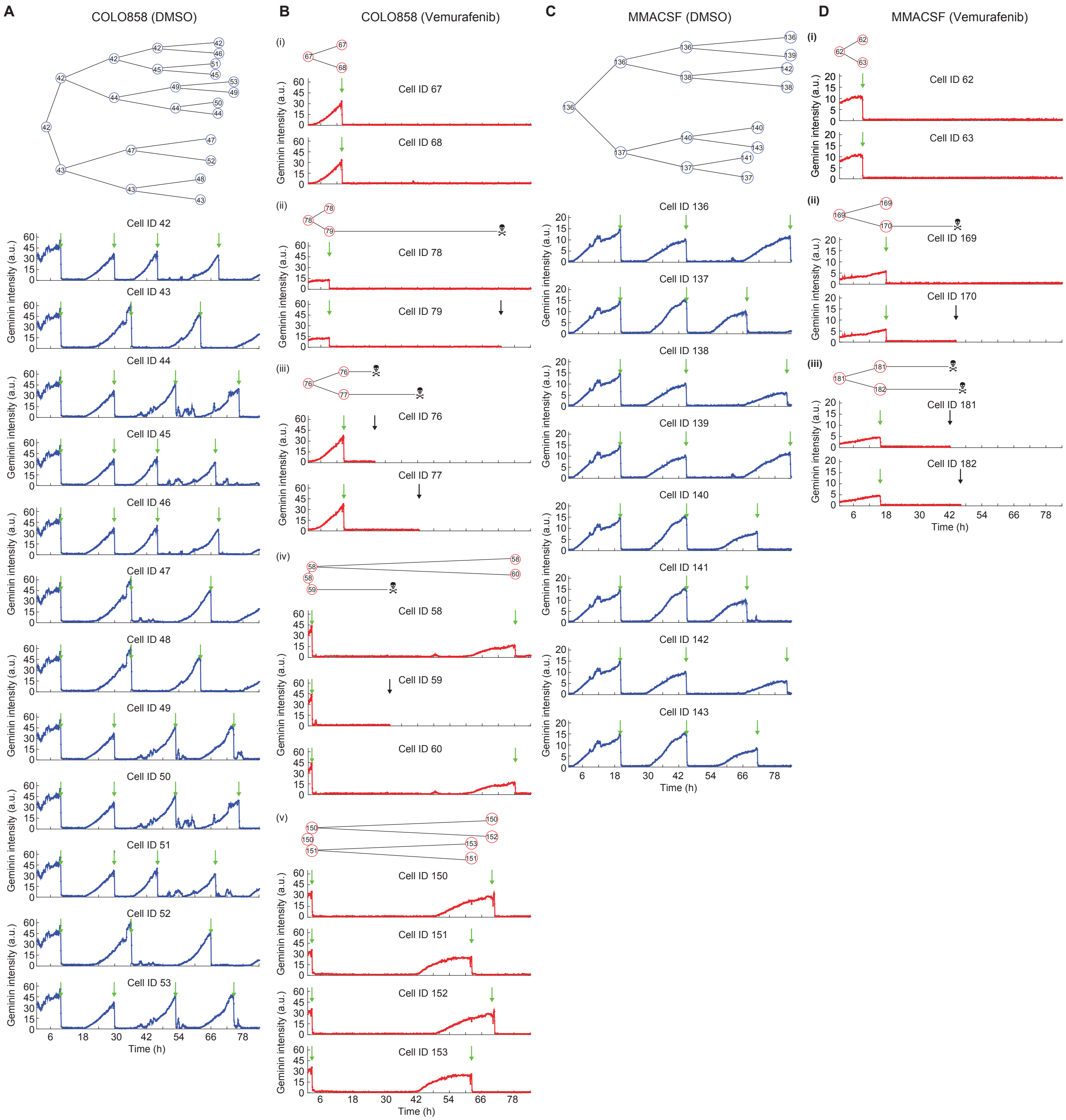

Appendix Figure S1. A slowly-cycling drug-resistant state involved in adaptation to RAF inhibitors. (A-D) Representative single-cell tracks for mCherry-geminin expression in COLO858 and MMACSF cells under different treatment conditions. Vemurafenib was used at 1 µM. Green and black arrows indicate cell division and cell death, respectively. A map of the cell lineage (on top) displays the relation between sister cells. Tracks for cells sharing a common parental cell show identical signals for mCherry-geminin as well as cell division events. Manually recorded cell divisions correlate with a sudden decrease in mCherry-geminin expression.

Appendix Figure S2

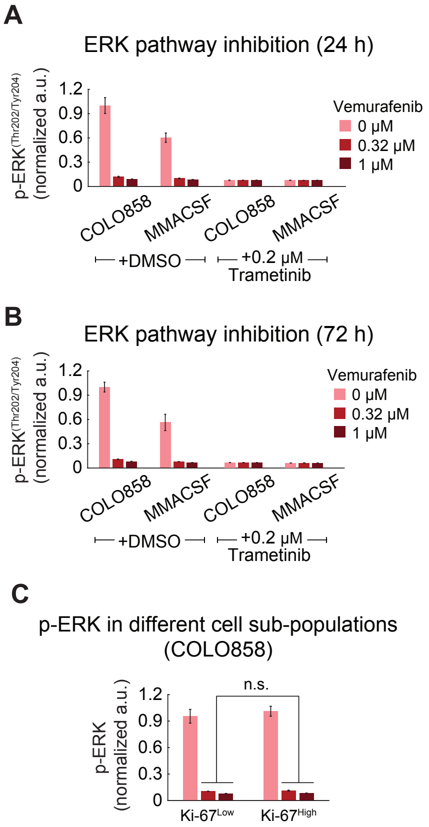

Appendix Figure S2. Drug adaptation is not explained by MAPK pathway reactivation. (A,B) p-ERK$^{T202/Y204}$ levels as measured in duplicate by immunofluorescence in COLO858 and MMACSF cells treated for 24 h (A) or 72 h (B) with vemurafenib in combination with DMSO or trametinib at indicated doses. (C) Mean p-ERK levels in Ki-67^{High} versus Ki-67^{Low} sub-populations of COLO858 cells treated with indicated doses of vemurafenib for 72 h. All data in (A, B, C) are normalized to DMSO-treated COLO858 cells at each time-point.

Appendix Figure S3

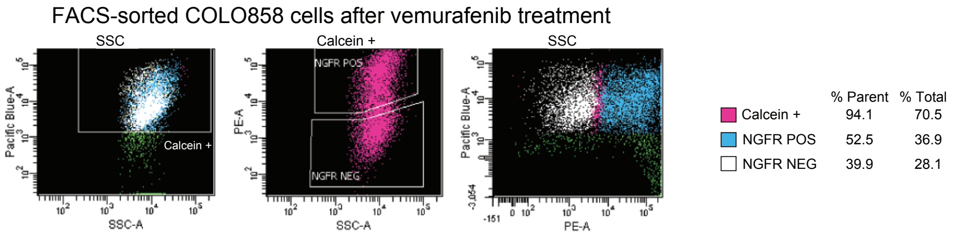

Appendix Figure S3. Fluorescence activated cell sorting of vemurafenib-mediated NGFR^{High} and NGFR^{Low} cell subpopulations. COLO858 cells exposed to 0.32 μM vemurafenib for 48 h were FACS-sorted to obtain NGFR^{Low} and NGFR^{High} sub-populations. Cells high for calcein-AM (viability marker) were gated and sorted to generate ~1 million NGFR^{High} and ~1 million NGFR^{Low} cells for further analysis.

Appendix Figure S4

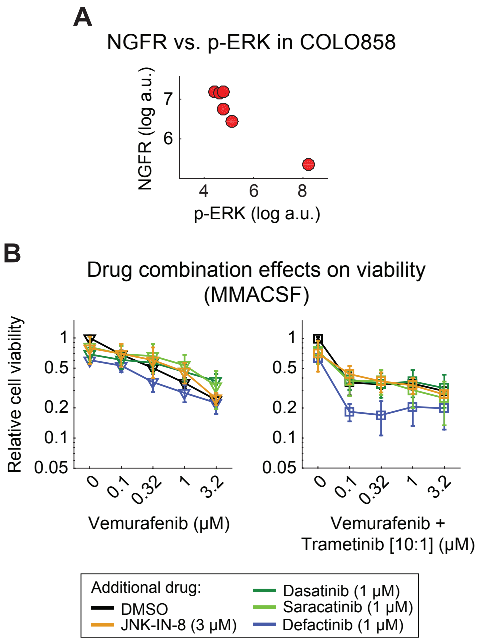

Appendix Figure S4. Concurrent inhibition of RAF/MEK signaling and the c-Jun/FAK/Src cascade overcomes vemurafenib resistance in NGFR^{High} cells. (A) Relationship between p-ERK$^{T202/Y204}$ and NGFR levels after 48 h treatment of COLO858 cells with combinations of different doses of vemurafenib (0, 0.32, 1 μM) and trametinib (0, 0.2 μM). (B) Relative viability of MMACSF cells treated for 72 h with vemurafenib or vemurafenib plus trametinib (10:1 dose ratio) in combination with DMSO, JNK-IN-8, dasatinib, saracatinib, and defactinib at indicated doses. Viability data was measured in 3 replicates and normalized to DMSO-treated controls.

Appendix Figure S5

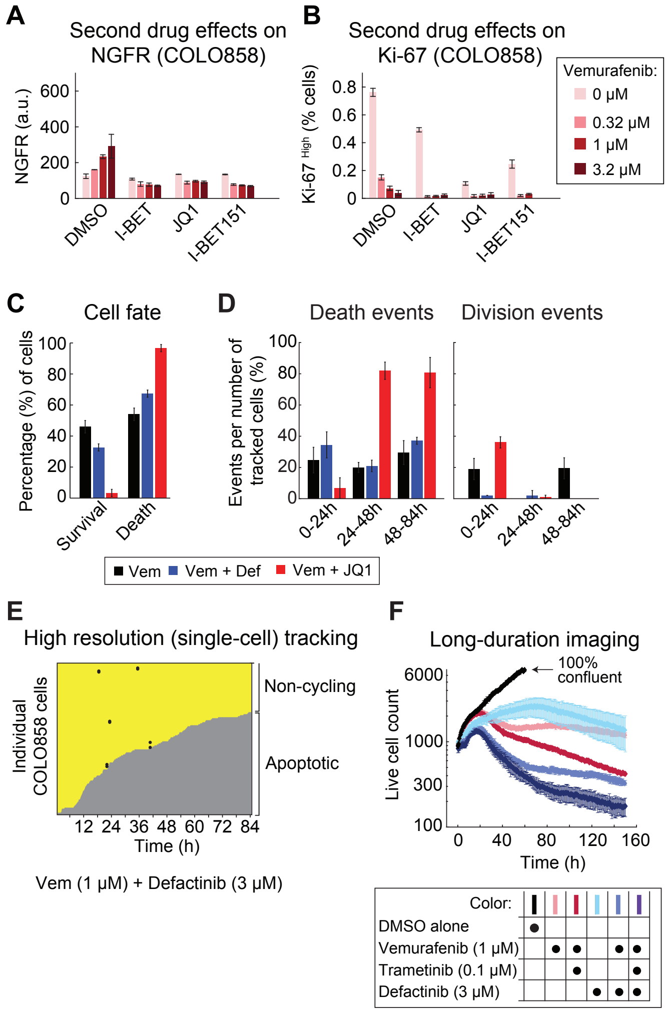

Appendix Figure S5. Suppression of the vemurafenib-induced NGFR^{High} state and slowly-dividing drug-resistant cells with BET inhibitors and other drug combinations. (A,B) NGFR protein levels (A) and percentage of Ki-67^{High} cells (B) measured by immunofluorescence in duplicate in COLO858 cells treated for 48 h with indicated doses of vemurafenib, in combination with DMSO, I-BET (1 µM), (+)-JQ1 (1 µM), or I-BET151 (1 µM). (C,D) Single-cell analysis following live-cell imaging of COLO858 cells treated with 1 µM vemurafenib in combination with DMSO, defactinib (3 µM), or (+)-JQ1 (0.32 µM) for 84 h. (C) Percentage of surviving and dead cells among cells tracked for 84 h. (D) Percentage of death and division events among cells tracked during indicated time intervals. (E) Single-cell analysis of division and death events following live-cell imaging of COLO858 cells treated with 1 µM vemurafenib in combination with DMSO or defactinib (3 µM) for 84 h. Data are presented as described in Figure 1. (F) Time-lapse analysis of COLO858 cells treated in 3 replicates for ~1 week with different drug combinations at indicated doses. Data for DMSO-treated cells are shown until day 3, the time at which cells reach ~100% confluency.

Appendix Figure S6

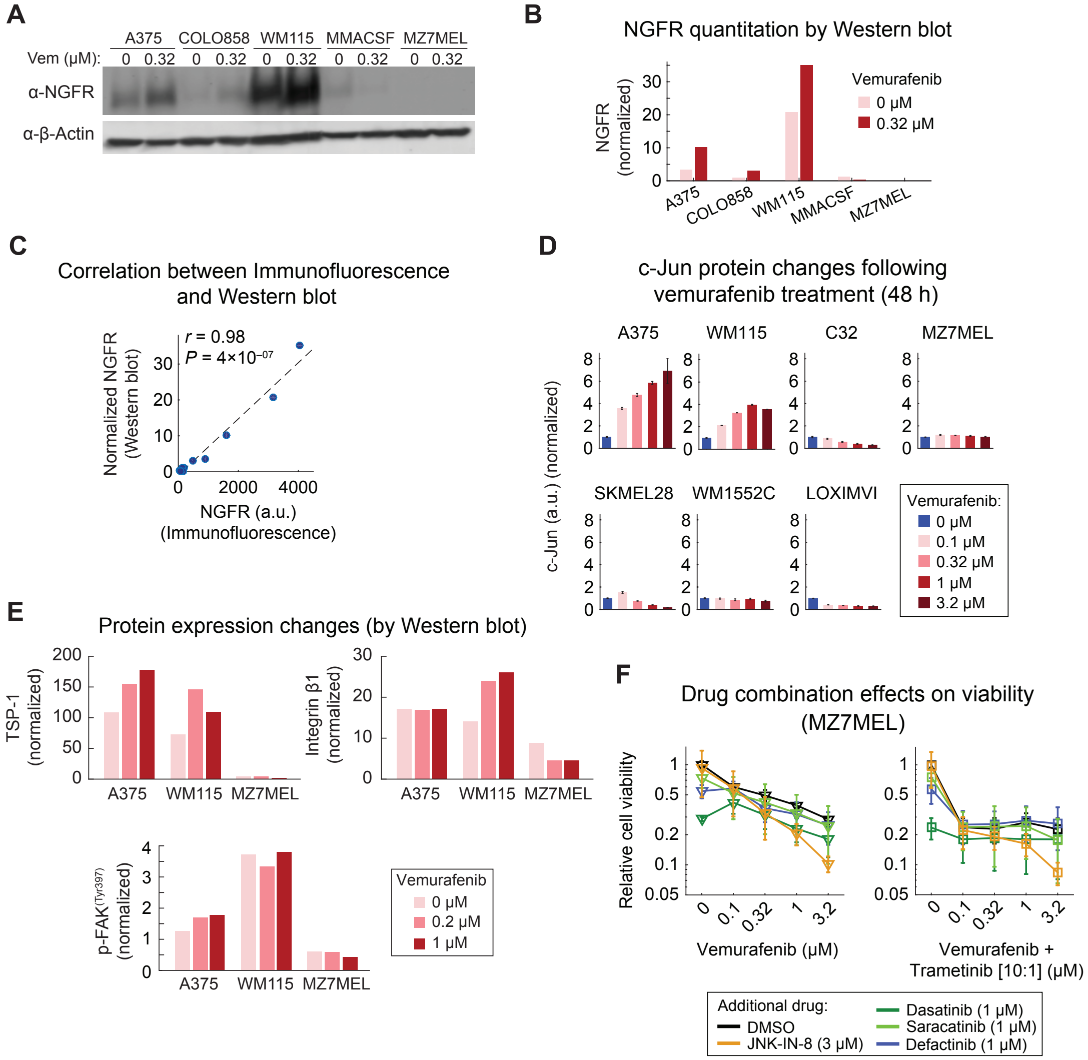

Appendix Figure S6. Inhibitors of JNK, FAK and Src kinases overcome the NGFR^{High} state in additional *BRAF*$^{V600E/D}$ melanoma lines. (A) Western blotting for COLO858, A375, WM115, MMACSF and MZ7MEL cells treated for 48 h with 0.32 μM vemurafenib or DMSO. (B) Quantified Western blot measurements for NGFR. Data are first normalized to β-actin levels in each cell line at each treatment condition and then to DMSO-treated COLO858 cells. (C) Pearson correlation between NGFR protein levels measured by immunofluorescence and Western blotting. (D) BRAF$^{V600E/D}$ melanoma cell lines were treated in duplicate with five doses of vemurafenib (0-3.2 μM) for 48 h. c-Jun protein levels were then measured by immunofluorescence. Data for each condition were normalized to DMSO-treated controls. (E) Quantified Western blot measurements for thrombospondin-1 (THBS1; TSP-1), Integrin β1 and p-FAK^{Y397} in A375, WM115 and MZ7MEL cells treated for 48 h with indicated doses of vemurafenib. Data are first normalized to HSP90α/β levels in each cell line at each treatment condition and then to DMSO-treated COLO858 cells. (F) Relative viability of MZ7MEL cells treated for 72 h with vemurafenib or vemurafenib plus trametinib (10:1 dose ratio) in combination with DMSO, JNK-IN-8, dasatinib, saracatinib, and defactinib at indicated doses. Viability data was measured in 3 replicates and normalized to DMSO-treated controls.

Appendix Figure S7

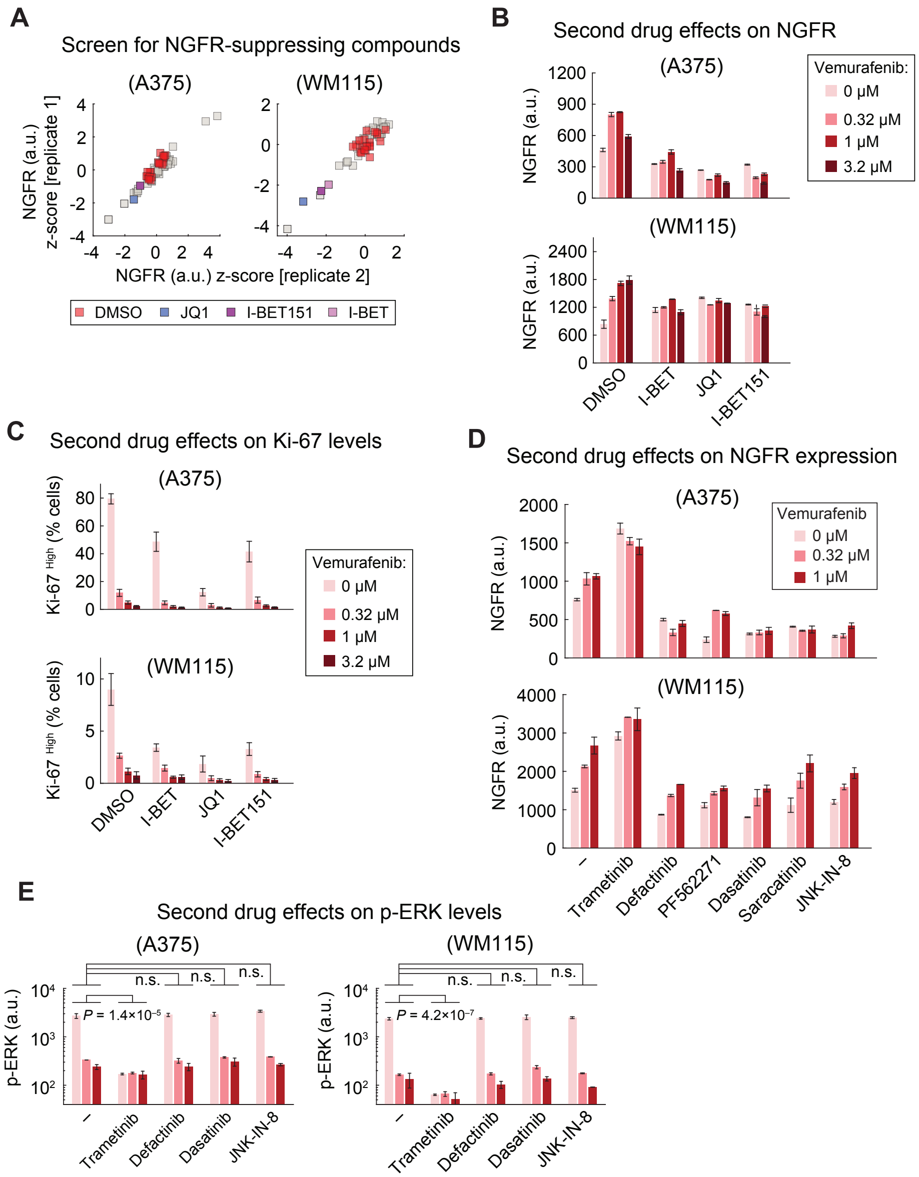

Appendix Figure S7. BET inhibitors, and JNK, FAK, and Src inhibitors suppress the NGFR^{High} state in additional *BRAF*$^{V600E/D}$ melanoma lines. (A) A375 and WM115 cells were treated for 48 h in duplicate with vemurafenib (at 1 μM) in combination with DMSO or three doses (0.11, 0.53 and 2.67 μM) of each of 41 compounds in a chromatin-targeting library. NGFR protein levels were measured by immunofluorescence, averaged across three doses of each compound, and z-scored. (B,C) NGFR protein levels (B), and percentage of Ki-67^{High} cells (C) measured by immunofluorescence in duplicate in A375 and WM115 cells treated for 48 h with indicated doses of vemurafenib, in combination with DMSO, I-BET (1 μM), (+)-JQ1 (1 μM), or I-BET151 (1 μM). (D) NGFR protein levels measured by immunofluorescence in duplicate in A375 and WM115 cells treated for 48 h with indicated doses of vemurafenib, in combination with DMSO, MEK inhibitor trametinib (0.6 μM), FAK inhibitors defactinib (3 μM) and PF562271 (3 μM), JNK inhibitor JNK-IN-8 (3 μM), or Src inhibitors dasatinib (3 μM) and saracatinib (3 μM). (E) p-ERK$^{T202/Y204}$ levels measured by immunofluorescence in duplicate in A375 and WM115 cells treated for 48 h with vemurafenib, in combination with DMSO or trametinib (0.2 μM), defactinib (3 μM), dasatinib (3 μM) and JNK-IN-8 (3 μM).

Appendix Figure S8

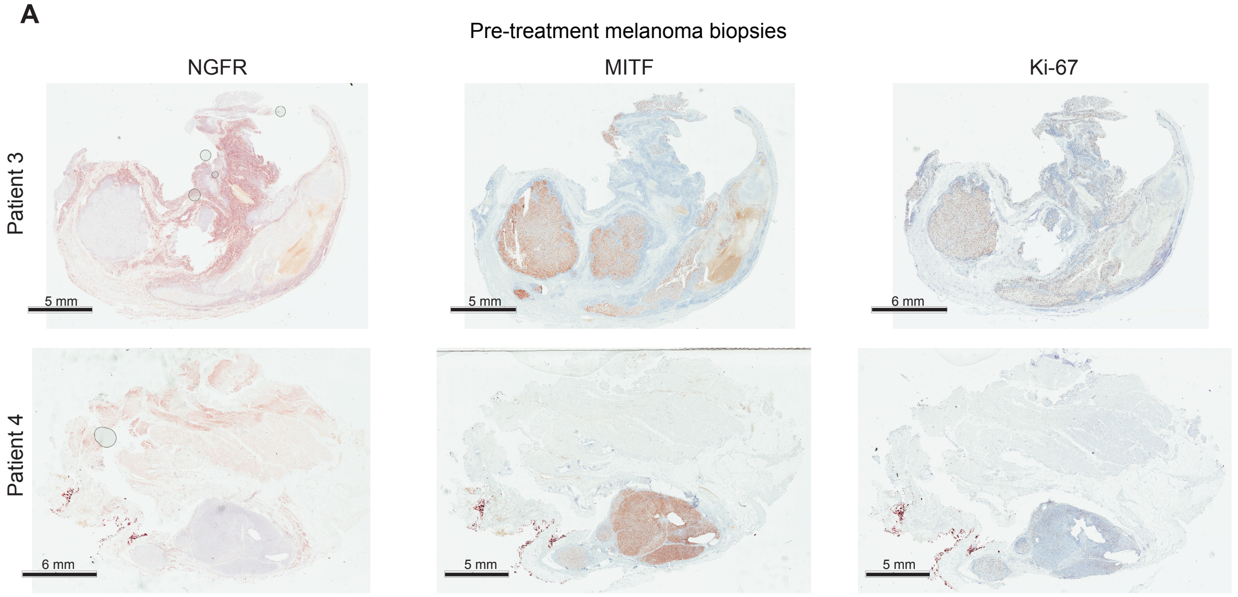

Appendix Figure S8. The NGFR^{High} state is tumor biopsies from melanoma patients. Immunohistochemical analysis of vemurafenib-naïve tumors from two melanoma patients staining for NGFR, MITF and Ki-67 (see Methods for patient clinical information).
