# Supplementary material for: Adaptive resistance of melanoma cells to RAF inhibition via reversible induction of a slowly dividing de‐differentiated state
Source: Mol Syst Biol. 2017 Jan 9;13(1):905. doi: 10.15252/msb.20166796 (PMC5248573; doi:10.15252/msb.20166796)
Supplement: Supplementary file 2 — Expanded View Figures PDF [file MSB-13-905-s002.pdf]

Expanded View Figures

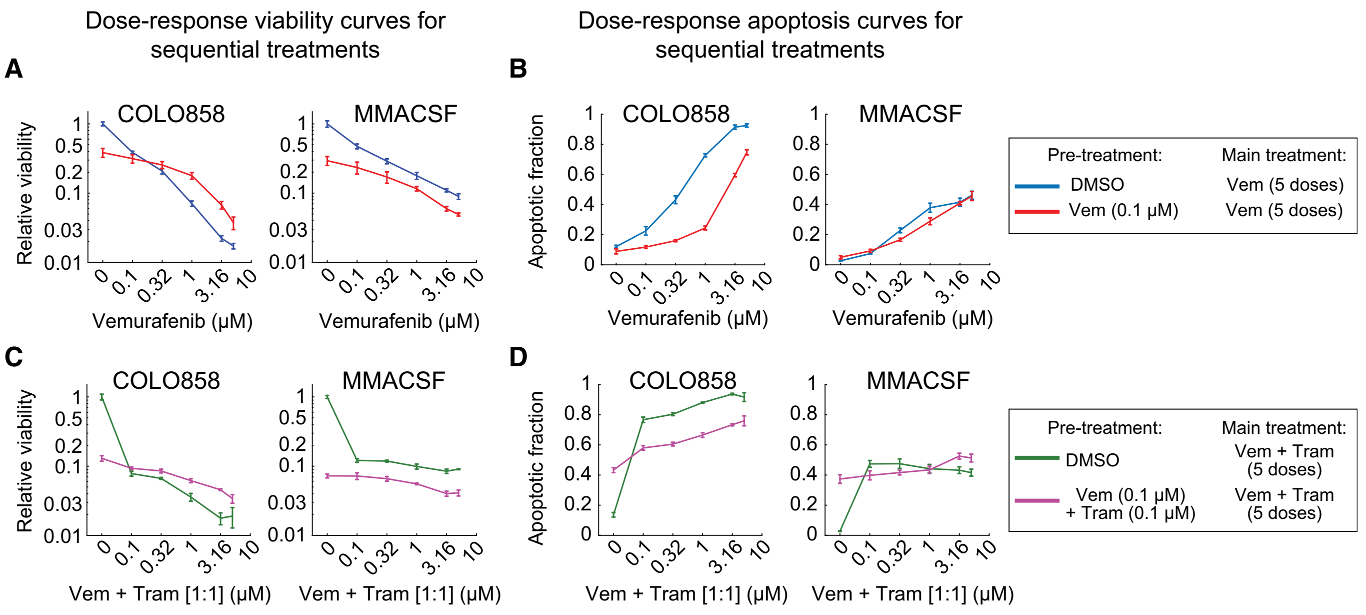

Figure EV1. Sequential drug treatments reveal adaptive resistance to RAF and MEK inhibitors.

A, B Cells were pre-treated for 24 h with DMSO or 0.1 μM vemurafenib and then treated with 0–5 μM vemurafenib for 72 h. Cell viability (normalized to the viability of cells pre-treated and treated with DMSO) (A) and apoptotic fraction of cells (B) were measured in four replicates. Data are presented as mean ± SD.

C, D Sequential treatment and measurements were repeated as described in (A, B) using vemurafenib in combination with trametinib with 1:1 dose ratio.

© 2017 The Authors

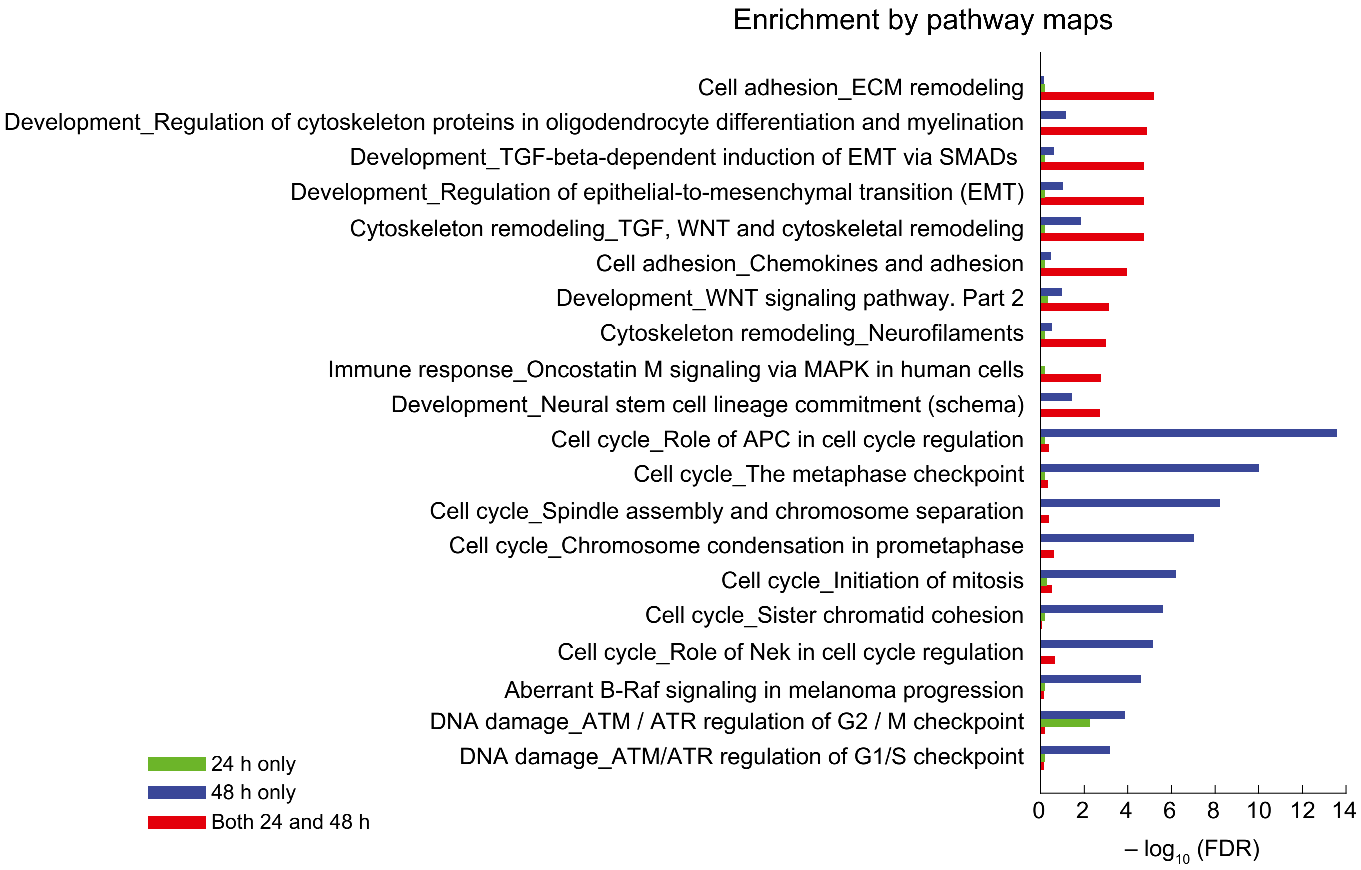

Figure EV2. Adaptive resistance to vemurafenib is associated with extracellular matrix (ECM) remodeling and cell adhesion pathways.
Top pathways differentially regulated between COLO858 and MMACSF cells treated with 0.2 µM vemurafenib for 24 and 48 h.

 © 2017 The Authors

A

Predicted transcription factors - receptors and secreted factors

Transcription factor	Count	Adjusted *P* value
ZIC3	79	1.01E-06
BACH1	92	3.44E-06
AP1	100	8.40E-05
NF1	71	8.31E-05
BPTF	84	9.94E-05
HMX1	81	1.21E-04
NFE2	67	1.62E-04
NFKAPPAB	66	2.87E-04
BACH2	76	3.18E-04
FOXD1	79	4.41E-04

B

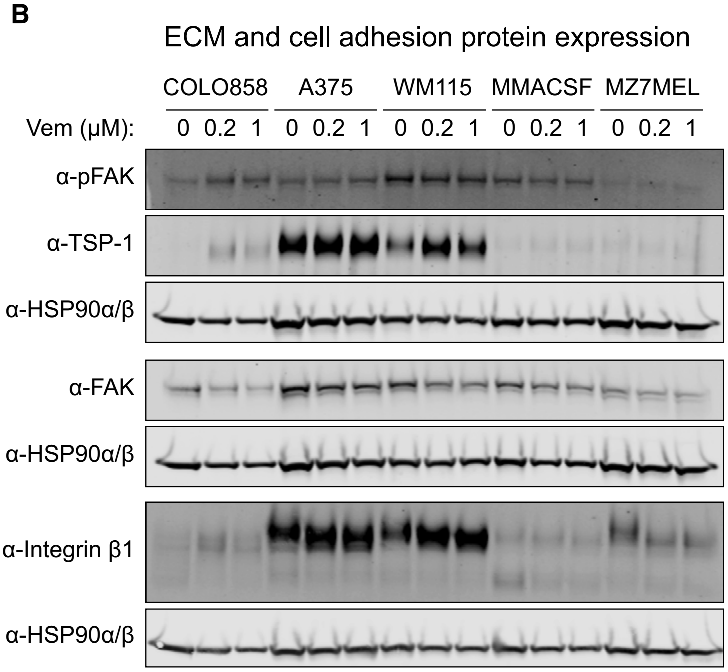

C

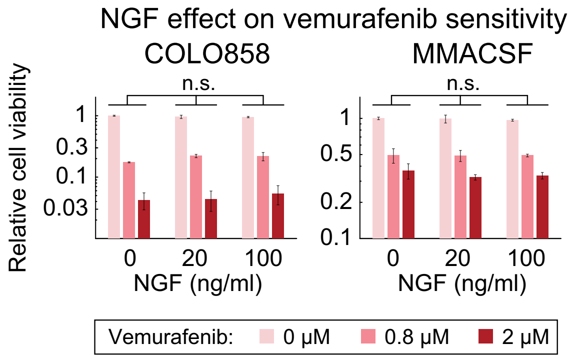

Figure EV3. The NGFR^{High} drug-resistant state is dependent on AP1 and focal adhesion signaling, but not NGF signaling.

A A list of transcription factor candidates predicted to regulate differentially expressed receptors and secreted factors between vemurafenib-treated COLO858 and MMACSF cells.

B Western blotting for NGFR-inducible COLO858 cells, NGFR^{High} A375 and WM115 cells, and NGFR^{Low} MMACSF and MZ7MEL cells, treated for 48 h with 0.2 or 1 µM vemurafenib or DMSO.

C The effect of NGF at indicated concentrations on viability of COLO858 and MMACSF cells treated in duplicate with vemurafenib at indicated doses for 48 h. Data are presented as mean ± SD. Statistical significance was determined by two-way ANOVA.

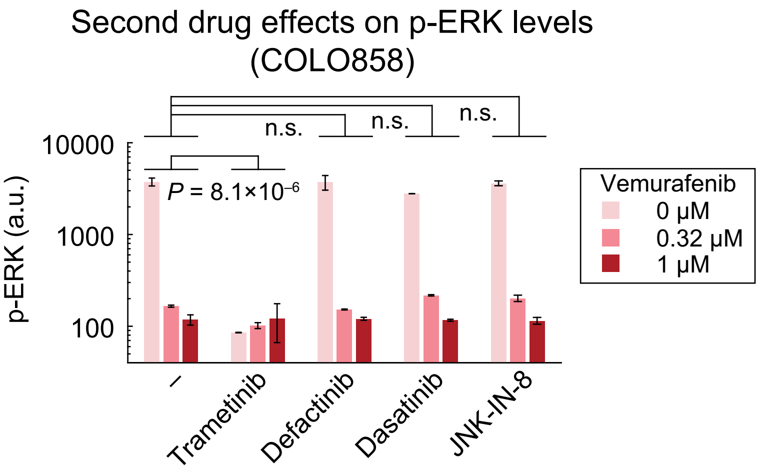

Figure EV4. The effect of drug combinations on ERK pathway activity.

p-ERK$^{T202/Y204}$ levels measured by immunofluorescence in duplicate in COLO858 cells treated for 48 h with vemurafenib, in combination with DMSO or trametinib (0.2 µM), defactinib (3 µM), dasatinib (3 µM), and JNK-IN-8 (3 µM). Data are presented as mean ± SD. Statistical significance was determined by two-way ANOVA.

© 2017 The Authors

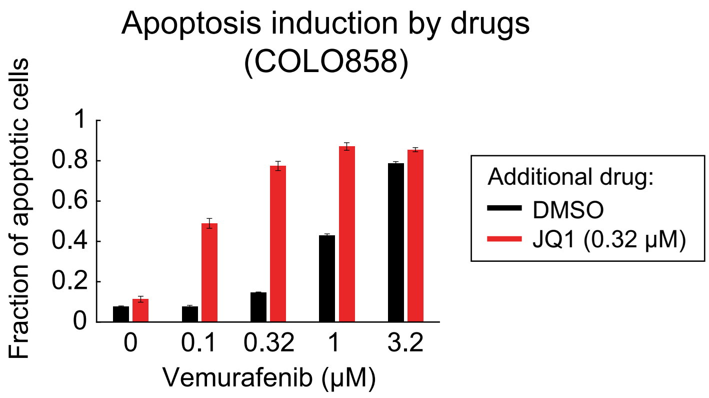

Figure EV5. Apoptotic response of COLO858 cells treated with JQ1 and vemurafenib.
Cells were treated for 72 h in three replicates with DMSO, or indicated doses of (+)-JQ1 and vemurafenib, alone or in combination. Data are presented as mean ± SD.

 © 2017 The Authors
